# Progress in Gene Therapy to Prevent Retinal Ganglion Cell Loss in Glaucoma and Leber's Hereditary Optic Neuropathy

**DOI:** 10.1155/2018/7108948

**Published:** 2018-05-02

**Authors:** Sara E. Ratican, Andrew Osborne, Keith R. Martin

**Affiliations:** ^1^John van Geest Centre for Brain Repair, Department of Clinical Neurosciences, University of Cambridge, Cambridge, UK; ^2^Geisel School of Medicine, Dartmouth College, Hanover, NH, USA; ^3^Eye Department, Addenbrooke's Hospital, Cambridge, UK; ^4^Cambridge NIHR Biomedical Research Centre, Cambridge, UK; ^5^Wellcome Trust-MRC Cambridge Stem Cell Institute, University of Cambridge, Cambridge, UK

## Abstract

The eye is at the forefront of the application of gene therapy techniques to medicine. In the United States, a gene therapy treatment for Leber's congenital amaurosis, a rare inherited retinal disease, recently became the first gene therapy to be approved by the FDA for the treatment of disease caused by mutations in a specific gene. Phase III clinical trials of gene therapy for other single-gene defect diseases of the retina and optic nerve are also currently underway. However, for optic nerve diseases not caused by single-gene defects, gene therapy strategies are likely to focus on slowing or preventing neuronal death through the expression of neuroprotective agents. In addition to these strategies, there has also been recent interest in the potential use of precise genome editing techniques to treat ocular disease. This review focuses on recent developments in gene therapy techniques for the treatment of glaucoma and Leber's hereditary optic neuropathy (LHON). We discuss recent successes in clinical trials for the treatment of LHON using gene supplementation therapy, promising neuroprotective strategies that have been employed in animal models of glaucoma and the potential use of genome editing techniques in treating optic nerve disease.

## 1. Introduction

In the last two decades, human gene therapy has advanced significantly. As our understanding of optic nerve disease pathologies has improved, the eye has become a particularly appealing clinical target for gene therapy and genome editing studies. The eye as a model organ for testing gene therapies has always been attractive. Firstly, the eye is largely immune privileged [1, 2] and vectors delivered to the eye are relatively isolated from the rest of the body. The size and ease of access to the eye is also favourable allowing small volumes of drug to be precisely delivered. Furthermore, testing the effectiveness of treatments via both electrophysiological and psychophysical testing is well-established, quick, and reliable [3]. Both Leber's hereditary optic neuropathy (LHON) and glaucoma are diseases resulting in permanent, irreversible loss of vision. With an undeniable need to treat and manage these diseases with novel therapeutic approaches, gene therapy may hold significant benefits in the coming years [4].

A prerequisite for a successful gene therapy in the eye is a vector system that leads to long, sustained levels of therapeutic gene expression within a select target cell with minimal side effects [5, 6]. This is particularly important for chronic, long-term pathologies such as glaucoma. Of the various nonviral and viral vector systems that have been used for retinal gene therapy (i.e., adenovirus, lentivirus, and nanoparticles), recombinant adeno-associated viral vectors (AAVs) have proven the most promising. Hundreds of clinical trials have been performed using AAV vectors spanning back 25 years [7] with 25 trials currently registered to test AAVs in retinal diseases (http://clinicaltrials.gov).

AAVs are composed of a 4.7 kb single-stranded genome packaged within a nonenveloped icosahedral capsid. After removal of 4.4 kb of the viral genome, vectors can be packaged with a similar-sized foreign piece of DNA that can be adjusted and optimized to improve cell-specific targeting and transgene expression via a series of cassette elements [8]. Various AAV serotypes exist whereby the vector capsid determines tissue specificity, with AAV2 regarded as the most efficient serotype for RGC transduction [9, 10]. In particular, vectors possessing an AAV2 backbone, cross packed into capsids from another serotype such as AAV2/2 and AAV2/6, have been shown to have the highest transduction efficiency in the retina [9]. AAV2/2 has been shown to transduce RGC more than any other cell type, while AAV2/6 displays the most diverse tropism profile, transducing Müller glia and many neuronal cell types [9].

**Figure 1:**
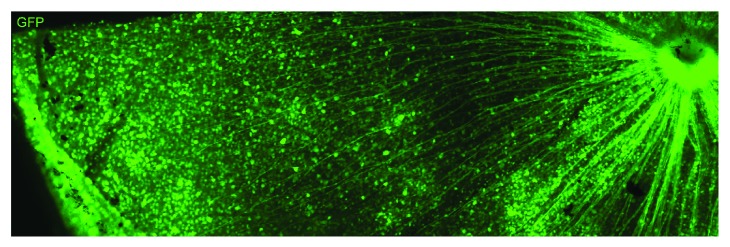
GFP expression throughout the mouse retina three weeks after intravitreal injection of AAV2-CAG GFP. Image courtesy of Dr. Andrew Osborne and Dr. Tasneem Khatib.

Promoter choice is also important for cell-specific expression and the strength of transgene expression within the targeted cell. While several RGC-specific promoters exist (Table 1), many vectors designed for optic neuropathies employ a ubiquitous CMV [10–14] or a hybrid CMV early enhancer/chicken b-actin promoter (CAG) [15–17] due to their small size and high levels of transgene expression [18, 19]. Promoter size is particularly important when looking to incorporate larger genes into an AAV. For optic neuropathies in particular, there is a reduced necessity for a selective promoter due to the method of delivery into the eye, which is a compartmentalized structure. The largely cell void vitreous means that the first cells which the vector particles come into contact with are of the inner retina, leading to a significant transduction of RGCs in the presence of an appropriate promoter. CMV and CAG promoters have been shown to facilitate transduction of around 85% of RGCs in the adult rat eye [10, 16] with similar expression seen in mice (Figure 1).

**Figure 2:**
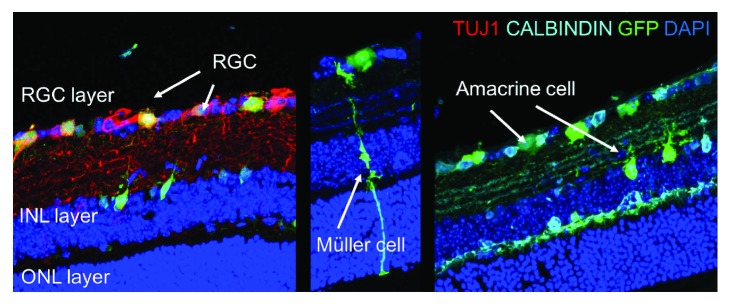
GFP expression and colocalization with retinal markers three weeks after intravitreal injection of AAV2-CAG GFP into the mouse eye. TUJ1 = retinal ganglion cells; CALBINDIN = amacrine cells. Image courtesy of Dr. Andrew Osborne.

Numerous studies support this observation, with both CMV and CAG promoters increasing expression primarily in RGCs, although some amacrine, Müller glial, and bipolar cells are also transduced [10, 14, 25] (Figure 2). Interestingly, a different profile of transduction is observed when the AAV2-CMV vector is injected at birth as opposed to adulthood. When intravitreally injected at P0, transduction is predominantly observed on rat photoreceptors (50%) compared to largely RGCs (60–70%) if injected in adults [10].

Depending on the gene therapy product and expected level of expression, careful consideration needs to be taken to ensure that off-target transduction does not have adverse effects on retinal function and health.

There is some limitation to AAV therapies, in particular the relatively small cargo capacity of AAV vectors for foreign DNA. Therefore, large genes are not suitable for use in a standard AAV vector, although dual vector [26] and more recently triple vector [27] approaches are being designed to overcome the coding capacity. Splitting large genes into two halves and packing them into two independent AAV vectors has made it possible to treat mouse models of Stargardt's disease and Usher syndrome type IB whereby the full-length, large gene is reconstituted in photoreceptors or the retinal pigment epithelium via splicing or homologous recombination [26]. Admittedly, this method is less effective than single AAV-mediated gene delivery and at present has not been used to address any genetic defects for glaucoma or LHON, but it removes a significant hurdle in the possible advancement of larger gene therapies.

For inherited optic nerve diseases where a specific genetic defect has been well defined, gene supplementation therapies have gained considerable ground. Recently, the U.S. FDA approved the first gene therapy for vision loss, created by Spark Therapeutics Inc., who utilized this approach in clinical trials for Leber's congenital amaurosis (LCA), a disease characterized by severe childhood visual loss. In their studies carried out by Bennett et al. [28], AAV-meditated gene supplementation was used to compensate for a deficiency in retinal pigment epithelium, caused by a mutation in the RPE65 gene. One year following phase 3 trials, the mean bilateral multiluminance mobility test (MLMT) change score was 1.8 (SD 1.1) light levels in the intervention group and only 0.2 (1.0) in the control group [29]. The results of this trial mark a significant improvement in functional vision in patients with RPE65 mutations, a condition that was previously medically untreatable.

While still unproven, rapid advances in precise genome editing technologies have provided the possibility of new gene therapy approaches to optic nerve diseases that have a clear genetic basis. RNA-guided nucleases (i.e., CRISPR/Cas9) and designer endonucleases (i.e., TALENs or ZFNs) [30] have been of particular interest and over the coming decade are likely to replace many of the current gene supplementation methods.

While still early in its development, CRISPR/Cas9-based therapies have already been demonstrated for the treatment of optic diseases [31, 32]. With this technique, the CRISPR-associated protein Cas9 creates site-specific double-stranded breaks in DNA [33], which can stimulate host DNA repair mechanisms [34]. In its most widely used form, this system requires that two components are expressed in cells: the Cas9 nuclease and a guide RNA (gRNA), consisting of a fusion of a crRNA and a fixed tracrRNA. The first 20 nucleotides of the gRNA correspond to the DNA sequence targeted for editing and direct Cas9 to this site using standard RNA–DNA complementarity base-pairing [35]. Gene knockouts can be created through nonhomologous end joining (NHEJ), which creates small nucleotide insertions or deletions (indels) resulting in a frameshift mutation and termination [36, 37]. Alternatively, site-specific integration of transgenes is typically achieved by homology-directed repair (HDR) in the presence of a donor DNA template [31]. While this pathway is not available to nondividing cells, a homology-independent targeted integration (HITI) strategy has recently been developed which achieves robust DNA knock-in in both dividing and nondividing cells *in vitro* and *in vivo* [38].

CRISPR/Cas9 can be efficiently delivered to select cell populations in the eye using a dual AAV system. In a proof-of-concept study by Hung et al. [39], a dual AAV2 system was used to introduce CRISPR/Cas9 into mouse RGCs *in vivo* and achieve knockout of a YFP transgene. With this dual-vector system, one AAV2 delivered SpCas9, while the other contained a single guide RNA (sgRNA) against YFP, achieving a knockout rate of 84% in YFP-sgRNA-infected retinal cells. Similarly, Yu et al. [31]. delivered CRISPR/Cas9 using a dual AAV system to therapeutically target the *Nrl* gene in postmitotic photoreceptors in mice. Deep sequencing of the targeted region indicated that 98% of total reads included changes almost exclusively at the targeted genome site.

Before CRISPR/Cas9 approaches can be transitioned to clinical use, the frequency of off-target activity and modification remains a major concern that will need to be assessed [35]. Possible long-term consequences may include Cas9 activation or repression, and possible epigenetic editing, although while long-term AAV-mediated gene expression is beneficial for gene supplementation therapies, CRISPR/Cas9 only requires a short period of expression [40]. Studies by Chew et al. [41] found that the editing frequency of CRISPR/Cas9 and its off-target effects had dose dependency. It is therefore likely that continuous AAV expression could contribute to a rise in off-target modification and should be considered when assessing these therapies.

Conditions with an unclear or heterogeneous etiology, however, will still require a gene therapy such as AAV that focuses on enhancing survival of neurons by manipulating molecular pathways in the host cell rather than by correcting a primary genetic defect. The rest of this review will focus on current strategies for the optic neuropathies glaucoma and LHON with a focus on AAV-mediated protection and the possibility of genome editing as a potential future treatment.

## 2. Gene Therapy to Treat Adult-Onset Glaucoma

Glaucoma is a group of eye diseases characterized by progressive and irreversible degeneration of retinal ganglion cells whose axonal projections constitute the optic nerve [42–44]. It is currently the leading cause of irreversible blindness worldwide [45] and by the year 2020 is projected to affect more than 76 million people [46]. Currently, the standard clinical treatment for glaucoma is based solely on lowering the intraocular pressure (IOP) of those affected through pharmacology, laser treatment, or surgery. However, surgery and laser treatment carry risks and often require further intervention or a combinational approach supplementing with additive topical therapies throughout a patient's life [46]. The need for such regular treatment, often with multiple different eye drops administered several times per day, means patient compliance is a challenge, and even in those adhering to treatment, a significant fraction continues to experience progressive visual loss even after their IOP is reduced [47, 48]. Therefore, there exists a great deal of interest in new therapeutic approaches which can be offered as a single injection directly into the eye and that lead to long-lasting or permanent beneficial outcomes.

Much of adult glaucoma has an unclear, heterogeneous etiology involving multiple genetic factors, individual risk factors, and environmental factors [43, 46, 49]. For these reasons, gene therapies for adult-onset glaucoma have focused primarily on neuroprotection, which involves slowing the loss of RGCs by altering their physiology to limit the pathogenesis of the disease. This can be accomplished in two ways: (1) by enhancing the activity of innate survival pathways in RGC or (2) by inhibiting the progression of cell death.

### 2.1. Enhancing the Activity of Innate Survival Pathways

Neurotrophic factors are known to promote neuron survival through activation of prosurvival pathways or inhibition of default apoptotic pathways when a cell experiences pathophysiological stress [50–52]. During development of the central nervous system, immature neurons require trophic factors to survive, differentiate, and establish synaptic connections. To control these developmental processes, some neurotrophic factors are expressed in limited quantities by target tissues and only neurons exposed to optimal neurotrophic levels survive and establish synaptic connections [53]. Neurotrophic factors have also been studied as potential neuroprotective factors in neurodegenerative diseases, with brain-derived neurotrophic factor (BDNF) and ciliary-derived neurotrophic factor (CNTF) both having been shown to protect axotomized RGC [54–56] and RGC in animal models of glaucoma [16, 57–61].

The biological effects of neurotrophins are mediated by cell surface receptors. BDNF acts by binding to the receptor tropomyosin-related kinase B (TrkB), which stimulates multiple signalling pathways within RGC, including extracellular signal-regulated kinases 1/2 (Erk 1/2) and the phosphatidylinositol-3 kinase (PI3K)/Akt pathways [12, 44, 62]. These pathways have been shown to play a key role in promoting neuronal survival and regeneration. Additionally, RGC are trophically dependent on BDNF with BDNF strongly expressed in the superior colliculus [63, 64] and transported retrogradely by RGC axons to ganglion cell bodies in the retina during both development and into adult life [65–69]. There is also substantial evidence that depletion of BDNF plays a central role in the onset of glaucoma, with evidence for impaired retrograde BDNF transport found in experimental models of ocular dysfunction [58, 70, 71].

Research conducted over the past two decades has consistently shown that intraocular injection of BDNF protein or AAV-mediated BDNF expression provides a robust but temporary neuroprotective effect on RGC after optic nerve transection or crush [54–56] or following ablation of the superior colliculus [72]. While these results offer promise for the use of neuroprotective strategies in glaucoma, overexpression of BDNF can cause tachyphylaxis of the survival response through downregulation of TrkB and its subsequent degradation [73]. Studies by Cheng et al. [12] have also found that mRNA levels of TrkB were reduced to 40% of their normal level following optic nerve transection in rats, and more recently, Guo et al. [74] used RGC-enriched mRNA samples from glaucomatous retinas and demonstrated a 97% decrease in TrkB message indicating that supplementation alone may not be sufficient in the long term.

Interestingly, combining AAV-CMV TrkB transduction with an intraocular injection of exogenous BDNF *in vivo* showed markedly increased neuronal survival compared to expressing TrkB alone [12]. At 2 weeks after axotomy, AAV TrkB with BDNF protected 76% of RGCs, whereas independent administration of BDNF or AAV TrkB promoted 38% or 27% neuronal survival, respectively [12]. This would imply a gene therapy strategy to increase both ligand and receptor expression which may provide a more potent, long-term treatment.

CNTF, which is expressed in all retinal cell layers, has also been identified as a potential neuroprotective agent for glaucoma gene therapy. Intravitreal injection of CNTF and adenoviral-mediated CNTF expression has been shown to increase STAT3 in RGC, implicating the JAK–STAT pathways as the pathway responsible for its survival effect [75]. In a study by Leaver et al. [57], intravitreal injection of AAV-CAG CNTF significantly increased RGC survival at 7 weeks after optic nerve crush and regenerating axons were visible in the distal optic nerve. Another study by Pease et al. [59] also found that AAV-CAG CNTF reduced axonal loss by 15% compared to control groups following laser-induced IOP elevation [55]. A potential limitation of CNTF is that exogenous gene transfer of CNTF impairs visual function in a dose-dependent manner [76], adversely affecting photoreceptor function [77–79]. It has also been shown to cause increased aberrant dendritic growth and a significant reduction in the complexity of the RGC dendritic arbor in both transduced and nontransduced RGC populations [80, 81].

Neurotech Pharmaceuticals has recently commenced a phase 2 clinical for its NT-501 encapsulated cell therapy, based on the therapeutic benefits of CNTF (http://clinicaltrials.gov/ct2/show/NCT02862938). This experimental treatment for glaucoma consists of surgically implanting small capsules into the eye, which are filled with human cells modified to secrete a steady stream of CNTF. Subjects will be followed for two years following implantation and will give greater insight into the therapeutic potential of sustained CNTF signalling.

### 2.2. Strategies to Inhibit Cell Death Pathways

Other approaches to gene therapy for glaucoma have explored the therapeutic potential of antiapoptotic proteins that inhibit the progression of RGC apoptosis. RGC have been shown to die by apoptosis in both experimental [82] and human glaucoma [83]. Caspase activity is the final common element central to the implementation of apoptosis in RGC, making caspase inhibitors an appealing prospect for neuroprotective glaucoma therapies. Taking this approach, McKinnon et al. [84] injected an AAV-CAG vector expressing a known caspase inhibitor, baculoviral IAP repeat-containing protein-4 (BIRC4), into one eye on rat models of glaucoma. BIRC4 was shown to significantly promote RGC survival, although significant differences in IOP exposure among treatment groups were observed. However, it should be noted that apoptosis plays an important role in controlling cell populations throughout the body and inhibitors of apoptosis have had limited success as therapies to date.

Affected by many proapoptotic stimuli, mitochondria also play a key role in determining the cell's fate, making them important therapeutic targets [85]. Proteins of the Bcl-2 family have been of particular interest as important mediators of mitochondrial integrity. In particular, Bcl-XL has been shown to repress apoptosis through the sequestration of proapoptotic proteins [86]. Malik et al. [85] showed that AAV2-SYN1-mediated overexpression of Bcl-XL in RGC of adult rat retinas provided a significant neuroprotective affect to optic nerve transections. 94% of transduced RGC survived the lesion compared to 15% of control RGC, and after 8 weeks, 46% of Bcl-XL overexpressing RGC remained viable. Modest improvements have also been observed with AAV2 neuron-specific enolase overexpression of Bcl-2-associated athanogene-1 (BAG1), an Hsp70/Hsc70-binding protein, which has been shown to suppress apoptosis and enhance neuronal differentiation through interaction with the MAPK cascade [87].

It has also been suggested that RGC experience greater metabolic stress than other retinal neurons. A study by Williams et al. [88] hypothesized that RGC go through a period of mitochondrial stress and metabolite depletion, which causes them to undergo greater fatty acid metabolism. Fatty acid beta-oxidation in particular can increase free-radical generation leading to a greater consumption of NAD^+^ [89], reducing its levels and leaving RGC vulnerable to damage from elevated IOP. To test this theory, an NAD^+^ precursor nicotinamide (vitamin B3) was orally administered at a high dose (2000 mg/kg per day) to DBA/2J mouse models of chronic, age-related, inherited glaucoma. At this high dose, 93% of eyes tested did not develop glaucoma over the 12-month study. As a single-dose therapy, AAV2-CMV expressing Nmnat1, a terminal enzyme involved in the last step of NAD^+^ production, was administered to DBA/2J mice. This protein was expressed in 83% of RGC at 2 weeks, and its overexpression was thought to drive further NAD^+^ production, thus preventing axon and soma loss, preserving axon transport and electrical activity in RGC and preventing glaucomatous nerve damage in more than 70% of treated eyes [88]. While further studies are needed to validate these findings in other models, combining this single molecule supplement with current glaucoma treatment may have a substantial impact on preventing progressive RGC death in glaucoma and could demonstrate therapeutic potential in other age-related diseases.

While neuroprotective approaches have shown promise, further developments will need to be made before these gene therapies can reach clinical use. Due to the slow progression of glaucoma, prolonged gene expression may be necessary to produce significant clinical benefit. However, prolonged expressions of BDNF and CNTF have been shown to alter the dendritic structure of both transduced and nontransduced RGC populations [81], although the functional importance of these changes remains uncertain. Additionally, long-term AAV-mediated secretion of BDNF or CNTF has been shown to significantly change the expression of endogenous retinal genes in transduced and nontransduced retinal tissue [90]. An analysis of unfixed whole retinal tissue infected with either AAV2-BDNF-GFP or AAV2-CNTF-GFP showed that 56% of the 93 retinal genes tested had significantly altered expression compared to control AAV2-GFP retinas with greatest fold changes in the RGC layer. Three times as many genes were altered after receiving CNTF treatment in comparison to BDNF treatment [90]. Long-term studies will need to be carried out to account for potentially adverse effects resulting from extended manipulation of gene expression and alterations in endogenous gene expression.

## 3. Gene Therapy to Treat Early-Onset Glaucoma

Unlike most forms of adult-onset glaucoma, early-onset glaucoma more frequently has a single genetic cause. Myocilin- (MYOC-) dominant gain-of-function mutations have been reported in approximately 4% of all primary open-angle glaucoma (POAG) cases, but 10–33% of juvenile open-angle glaucoma cases harbour a MYOC mutation [91, 92]. While they do not directly disrupt RGC function, these mutations have been shown to affect trabecular meshwork (TM) functioning and lead to elevated IOP [93]. Mutations in the genes encoding cytochrome P450 have also been identified in 40% of individuals with primary congenital glaucoma [92, 94]. With advances in the specificity and efficiency of genome editing tools, there exists the possibility that these mutations can be targeted therapeutically. In a recent study by Jain et al. [91], adenoviruses expressing CRISPR/Cas9 components (Ad5-cas9 and Ad5-cr*MYOC*) were used to target dominant MYOC mutations in a mouse model of myocilin-associated POAG. Cas9 was shown to effectively disrupt the mutant MYOC gene, lowering IOP in treated mouse eyes and preventing further glaucomatous damage to RGC. In the same study, these constructs were used to treat trabecular meshwork tissue in human ex vivo cultured eyes [91]. A reduction of myocilin mRNA was observed, suggesting the feasibility of translating this technology to patients with MYOC mutations.

## 4. Gene Therapy to Treat Leber's Hereditary Optic Neuropathy

Leber's hereditary optic neuropathy (LHON) is an optic nerve disorder characterized by rapid, painless visual loss in one eye, shortly followed by successive visual loss in the remaining eye [95]. The disease is seen more predominantly in men, and symptoms appear in the second to third decades of life [65]. LHON is caused by point mutations in mitochondrial DNA (mtDNA). Three of the most common mutations, causing approximately 95% of LHON cases, lead to decreased NADH dehydrogenase activity in complex I of the mitochondrial respiratory chain and a reduction in energy production by the mitochondria [96–98]. In particular, mutation of the NADH dehydrogenase subunit 4 complex I (*ND4*) gene (G11778A) is present in 60% of LHON cases worldwide [99]. Though these genes are expressed in all mitochondria, the disease phenotype is limited to loss of RGC and degeneration of the optic nerve [100], possibly due to increased RGC energy demands. The majority of individuals with LHON are homoplasmic, meaning they carry only mutant mtDNA. Approximately 15% of LHON patients are heteroplasmic for the primary LHON mutation, with varying proportions of mutated and wild-type mtDNA present in peripheral blood leukocytes [101].

There is currently no definitive treatment for LHON. While current interventions of idebenone and vitamin B12 therapy have been effective in some patients, they have no effect in others [102]. Within the last decade, gene therapy research for LHON has advanced significantly, from the first LHON animal model by Qi et al. [103] to recent clinical trials supporting the feasibility of using gene therapy to treat this disorder through allotropic expression of the corrected mitochondrial gene sequences. The future treatment for LHON is likely to be through (1) incorporating new mitochondrial DNA via AAV or (2) CRISPR/Cas9 technology for mtDNA editing which may provide potentially novel avenues to treat heteroplasmic LHON mutation.

### 4.1. Incorporating Mitochondrial DNA via AAV

How LHON disease pathology develops is determined by the balance between the amounts of mutant and normal protein (typically ND4) expressed in RGC [100]. As a result, gene therapy studies have focused on promoting expression of the normal protein in a degree high enough to prevent RGC degeneration. Currently, viral vectors can only transfer genes to the nucleolus and not directly to mammalian mitochondria. To overcome this limitation, Guy et al. [104] developed what has become the foundation for allotropic expression of LHON mitochondrial genes. AAV-CAG were used to construct a nuclear version of the mitochondrial gene which codes for cytoplasmically expressed proteins. The proteins generated contain a mitochondrial targeting sequence, which allows for effective trafficking to their target site in the mitochondria [104]. This method was validated *in vivo* using a rat model of LHON, where electroporation of a construct carrying the wild-type ND4 allele effectively protected against RGC degeneration using allotropic expression [105].

Validating this method has led to rapid advances in LHON gene therapy. In 2015, Cwerman-Thibault et al. [106] introduced a recombinant AAV2 containing ND4 into a rat model of LHON, demonstrating both the safety and efficacy of allotropic ND4 expression in treating LHON. This approach suppressed RGC degeneration and preserved visual function in mutant ND4 rodents, making it an appealing candidate for human LHON clinical trials.

Recently, the results of the first three clinical trials of LHON gene therapy were released and provide promising prospects for the future. A study by Wan et al. [96] carried out at Tongji Hospital in Wuhan, China, evaluated the safety and efficacy of AAV2-mediated allotropic expression of ND4 in nine patients with the G11778A mutation. AAV-ND4 was injected in one eye each in each of nine patients, all of whom had been diagnosed for over a year. Each patient was followed for nine months. Over that period, none of the patients showed local or systemic adverse side effects from the treatment and visual acuity of 6 of the 9 patients showed significant improvement. In particular, patient 1 improved from 2 to 1.1 log MAR and was able to read newspaper headlines after treatment and patient 6 (a 9-year-old female) improved her visual acuity from 1.2 to 0.4 log MAR, allowing her to return to school and study with her classmates [96].

The results of phase I clinical trials performed in the United States by Guy et al. [107] also showed modest but statistically significant improvements of visual acuity following allotropic LHON gene therapy. 14 patients in three groups with varying durations of vision loss were recruited for this study, which administered low and medium doses of the gene therapy. Similar to the Chinese study, no serious safety concerns were associated with allotropic gene therapy of LHON [96, 107]. Following intravitreal injection, improvements in treated eyes were observed within 7 to 30 days [107], a duration when expression was observed in 90% of rodent RGC as reported by Koilkonda et al. [108]. High-dose cohorts are now being established for the next phase of clinical testing.

Similar to the previous studies, Vignal et al. [109] conducted a single-centre phase 1/2 clinical trial of rAAV2/2-ND4 that included 15 subjects and 4 dose-escalation cohorts (9 × 10^9^ vector genomes [vg]/eye, 3 × 10^10^ vg/eye, 9 × 10^10^ vg/eye, and 1.8 × 10^11^ vg/eye). The study found that rAAV2/2-ND4 was safe and well tolerated 2 years after a single intravitreal administration with an observed between-eye difference in visual acuity change from baseline noted at week 96, favouring the treated eye.

While still in its early stages, the findings of these studies support the development of further clinical studies on allotropic gene therapies for LHON, but with larger patient cohorts. With mild improvements reported, it is reasonable to assume that normal ND4 was effectively targeted to the mitochondria and was able to increase energy supply to the optic nerve in both studies [96, 107]. Initial findings suggest that the degree of response to treatment is highest when treatment is administered at 1 year of visual loss [107]. In future studies, the duration of visual loss and degree of remaining optic nerve function will be important considerations to determine the timing of administration and clinical efficacy of each LHON gene therapy.

### 4.2. CRISPR/Cas9 Gene Shifting

Gene shifting describes the process of selectively destroying mutant mtDNA while preserving normal mtDNA in heteroplasmic mitochondrial diseases, which make up approximately 15% of LHON cases [110]. As described previously, the development of LHON is the result of the balance between the amount of normal and mutant protein [100]. The majority of mtDNA mutations cause disease when the level of heteroplasmy exceeds 70–80% [111].

Advances in the CRISPR/Cas9 system for efficient genome editing in mammals have opened up new avenues for intervention of inherited diseases. Studies by Hung et al. [39] have demonstrated that CRISPR/Cas9 components can efficiently target RGCs and achieve high levels of gene knockout *in vivo* using a dual AAV2 system. While CRISPR/Cas9 has been widely used to edit nuclear DNA, it has been unclear whether this system could be applied to edit mtDNA, with the mitochondrial inner membrane presenting a substantial entry barrier to AAVs. In a recent study, Jo et al. [112] demonstrated that FLAG-Cas9 is able to localize to the mitochondria with sgRNAs to edit mitochondrial DNA in a site-specific manner. To overcome nonspecific distribution of FLAG-Cas9, a mitochondria-targeted Cas9 (mitoCas9) was also created which localizes only to the mitochondria to produce site-specific cleavage of mtDNA. While further validation of this approach and analysis of off-target editing would be necessary, advances in CRISPR/Cas9 technology for mtDNA editing presents an opportunity to study the effects of gene shifting through knockout of mtDNA. With continued advances in this technology, future studies on AAV-mediated genome editing in heteroplasmic LHON cases may be possible.

Taking a different approach, the concept of gene shifting could potentially be applied to reduce mutant mtDNA copies in the oocytes of women carrying LHON mutations. In a recent study by Reddy et al. [113], mitochondrial targeted endonucleases known as mitoTALENs were able to eliminate mutant mitochondrial haplotypes in both mice oocytes and one-cell embryos, leaving only the selected mtDNA haplotype. This heteroplasmy shift resulted in phenotypically normal animals and prevented transmission of the eliminated mtDNA haplotypes to future offspring [113]. Additionally, mitoTALENs targeting the 14459G>A human LHON mutation were able to successfully reduce mutant mtDNA in mouse oocytes 24 hours after infection [113].

While there is a long way to go in further developing these technologies, genome-editing techniques are advancing at an astonishing rate. The development of CRISPR/Cas9 in particular demonstrates several advantages in comparison to ZFNs or TALENs. CRISPR/Cas9 has been shown to be more efficient at inducing genetic modifications [114] and can be easily adapted to target different DNA sites requiring only a simple modification to the 20 bp protospacer region of the gRNA, whereas ZFNs and TALENs require recoding of proteins using large 500–1500 bp DNA segments [115]. Cas9 also has the unique advantage of being able to induce double-stranded breaks at numerous sites in parallel using multiple RNA guides [37]. Ensuring the elimination of off-target cleavage with these editing techniques remains a primary concern [35] and justifiable barrier to clinical use. Additional consideration is also needed regarding when or if these types of intervention are necessary. Only 50% of male and 10% of female patients with the G11778A LHON mutation actually develop visual loss [99]. While predictions of susceptibility to disease can be made, making unnecessary modifications could pose a significant risk to what might be otherwise healthy eyes.

## 5. Conclusion

Substantial developments in technology over the past two decades have made glaucoma and LHON exciting potential targets for gene therapy. While further trials with larger patient cohorts are needed, initial results of LHON gene therapy clinical trials demonstrate the progress made and prospects for future clinical use of gene therapy. Future research will need to determine the ideal timing of gene therapy administration and whether a “critical window” for effective treatment exists either before or after symptoms occur. While many hurdles will need to be overcome before glaucoma gene therapy treatments reach routine clinical use, our advances in understanding the disease's pathology and our achievements in RGC neuroprotection in animal models provide grounds for optimism. Moving forward, the most challenging aspect of translating these findings will be determining the best avenue for RGC neuroprotection. Several different animal models of glaucoma and optic nerve disease are currently used in research, with each having advantages and limitations. Clinically, the presentation of glaucoma pathology also varies significantly. Determining the most effective pathway to target will depend heavily on the specific features of each case. In some patients, multiple pathways may contribute to the onset of the disease. Nevertheless, these rapid developments and successes achieved thus far give reason to believe that novel clinical therapies are on the horizon.

## Figures and Tables

**Table 1:** Promoters typically chosen for transduction of retinal ganglion cells (RGCs) within the eye.

Promoter	Specificity for RGCs	Strength of expression in RGCs	Off-target labelling	Size (bp) % of AAV cargo	References
CMV	+	+++	Muller glia Amacrine cells Bipolar cells	(508–800) *(10–17%)*	[10–14]

CAG	+	++++	Muller glia Amacrine cells Bipolar cells	(584–1132) *(12–24%)*	[15–17]

SYN1	+++	++	Amacrine cells	(400–469) *(8–10%)*	[20, 21]

Nefh	*++++*	+++		(2251) *(48%)*	[22]

Thy1	*++++*	++		(6500) (exceeds limit)	[23]

Mcp-1	++	+++	injured cells	560 (mouse only) *(12%)*	[24]
